# (Not) Keeping the stem straight: a proteomic analysis of maritime pine seedlings undergoing phototropism and gravitropism

**DOI:** 10.1186/1471-2229-10-217

**Published:** 2010-10-06

**Authors:** Raul Herrera, Catherine Krier, Celine Lalanne, El Hadji Maodo Ba, Alexia Stokes, Franck Salin, Thierry Fourcaud, Stéphane Claverol, Christophe Plomion

**Affiliations:** 1 Instituto Biología Vegetal y Biotecnología, Universidad de Talca, 2 Norte 685, Talca, Chile; 2 INRA, UMR Biogeco 1202, 69 route d'Arcachon, 33612 Cestas, France; 3 Inspection Régionale des Eaux et Forêts de Kolda, Bp 57 Kolda, Senegal; 4 INRA, UMR AMAP, Montpellier 34000, France; 5 CIRAD, UMR AMAP, Montpellier 34000, France; 6 Pôle protéomique de la Plateforme Génomique Fonctionnelle Bordeaux, Université Bordeaux 2, Bordeaux, France

## Abstract

**Background:**

Plants are subjected to continuous stimuli from the environment and have evolved an ability to respond through various growth and development processes. Phototropism and gravitropism responses enable the plant to reorient with regard to light and gravity.

**Results:**

We quantified the speed of maritime pine seedlings to reorient with regard to light and gravity over 22 days. Seedlings were inclined at 15, 30 and 45 degrees with vertical plants as controls. A lateral light source illuminated the plants and stem movement over time was recorded. Depending on the initial angle of stem lean, the apical response to the lateral light source differed. In control and 15° inclined plants, the apex turned directly towards the light source after only 2 h. In plants inclined at 30° and 45°, the apex first reoriented in the vertical plane after 2 h, then turned towards the light source after 24 h. Two-dimensional gel electrophoresis coupled with mass spectrometry was then used to describe the molecular response of stem bending involved in photo- and gravi-tropism after 22 hr and 8 days of treatment. A total of 486 spots were quantitatively analyzed using image analysis software. Significant changes were determined in the protein accumulation of 68 protein spots. Early response gravitropic associated proteins were identified, which are known to function in energy related and primary metabolism. A group of thirty eight proteins were found to be involved in primary metabolism and energy related metabolic pathways. Degradation of Rubisco was implicated in some protein shifts.

**Conclusions:**

Our study demonstrates a rapid gravitropic response in apices of maritime pine seedlings inclined >30°. Little or no response was observed at the stem bases of the same plants. The primary gravitropic response is concomitant with a modification of the proteome, consisting of an over accumulation of energy and metabolism associated proteins, which may allow the stem to reorient rapidly after bending.

## Background

Plants have sophisticated mechanisms to interpret environmental stimuli so as to optimize resource allocation at any time [1]. Light, being indispensable for plant growth and photosynthesis, is an important factor that determines stem orientation. Plants can also sense gravity, which enables stems and branches to maintain their position with regard to a given axis [2]. Shoot orientation is therefore a result of the combined (either synergistically or antagonistically) effect of both negative gravitropism in response to gravity, and positive phototropism in response to light [3]. Little information exists concerning the interactions between these two dynamic processes in trees. The consequences of stem bending on wood quality can be major [4], and also reflected throughout a tree's life.

One of the earliest studies on gravitropism, carried out in the 19^th ^century [5], showed that plant shoots kept in the dark grew upwards. Therefore, light is not the sole reason for plants to grow vertically. The same results were found by Fukaki *et al. *[6], who repeated the experiment on *Arabidopsis thaliana*. However, due to the ubiquitous presence of gravity on earth, it has been difficult to separate the effect of both gravity and light on plant growth and to study their interaction with regard to stem directional growth. The use of clinostats [7], chronic centrifugation [8] or spaceflight [9-11], has allowed the study of shoot orientation in reduced or modified gravity. In most cases, shoots responded to microgravity (through vertical growth) but in each experiment, lighting was vertical, therefore the directions of gravity and light stimuli were parallel. Experiments in normal gravity where light exposure was unilateral have shown that the elongating apex grows towards the light [2,3]. This bending movement occurs due to changes in auxin gradients.

Most research on gravi- and photo-tropic responses has been carried out on annual plants, in particular oat (*Avena sativa *L), maize (*Zea mays *L.) and Arabidopsis seedlings [12-14]. Although the findings reported for these species are essential to understand how plants grow, trees may present an additional level of complexity in their response to gravity and light. In addition to the primary response to these stimuli, a secondary and irreversible response is typical to these long lived organisms: the formation of reaction wood. Reaction wood is formed on the underside of the leaning stem in conifers (called compression wood), and on the upper side in angiosperms (called tension wood). Reaction wood formation is a complex developmental process that enables tree stems and branches to reorient with regard to gravity, thus restoring a more favorable position in space and over time [4,15]. In stems, this reorientation can often be seen after a permanent displacement from the vertical has occurred e.g. after wind or snow loading. It has been claimed that gravity is the main force triggering stem reorientation and reaction wood formation [16]. However, in inclined *Quercus crispula *seedlings, Matsuzaki *et al. *[17] demonstrated, that unilateral light alone resulted in stem phototropism through asymmetric growth involving tension wood formation. Schamp *et al. *[18] also showed that phototropic bending occurred in the direction of greatest canopy openness in the main stems of three broadleaf species.

At the molecular level, our understanding of gravity and light perception and transduction pathways has greatly advanced due largely to studies on Arabidopsis mutants [3,19-24]. The use of these plants along with mutants possessing photoreceptor genes having abnormal responses to different exposures, types and intensities of light, has allowed the dissection of both types of tropisms [13,25,16]. Despite the existence of different perception mechanisms for gravity and light, some molecular components of both signal transduction stimuli may be common to both pathways e.g. ethylene, calcium, auxin and their receptors [3], while other components may differ.

**Figure 1:**
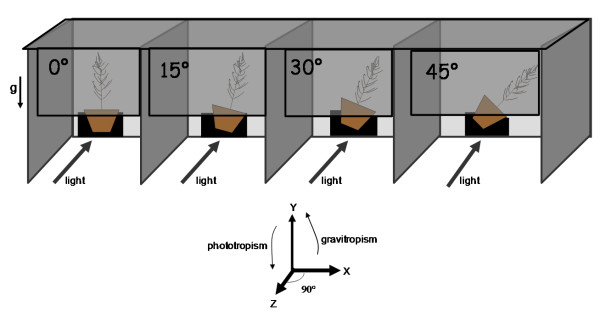
**Seedlings were either vertical (0°) or inclined along the x axis at 15°, 30° and 45°**. Illumination was provided by lights situated perpendicular (z axis) to the x axis. Each compartment housed one tree and was covered by black cardboard to prevent parasitic light reaching the plants. Stems reoriented with regard to the vertical (y) axis (gravitropism) and the horizontal (z) axis (phototropism).

In this context, the main objective of this study was to identify proteins responding to gravity and light in the apical shoot of maritime pine seedlings. This species is the most widely planted commercial forest tree in southwestern Europe. We designed an experiment whereby vertical (0°) and inclined plants (15°, 30° and 45° from the vertical) were illuminated unilaterally from a direction perpendicular to the inclination (Figure 1) for 22 d, allowing us to quantify the speed and intensity of gravi- and photo-tropism. We described the molecular response in the apical shoot after 22 hr and 8 days of treatment, by generating proteomic data using two-dimensional gel electrophoresis and tandem mass spectrometry. This experiment aimed at answering the following questions: Which stimulus is stronger: light or gravity? How quickly and at which intensity does the shoot of a maritime pine seedling respond to light and gravity? To what extent does this response depend on the leaning angle of the plant? What kinds of proteins are synthesized by the apical shoot of stimulated plants? Do the same proteins accumulate in the apical shoot in phototropic and/or gravitropic stimulated plants?

## Results

### Phototropic and gravitropic responses

#### Reorientation of the apical region

**Figure 2:**
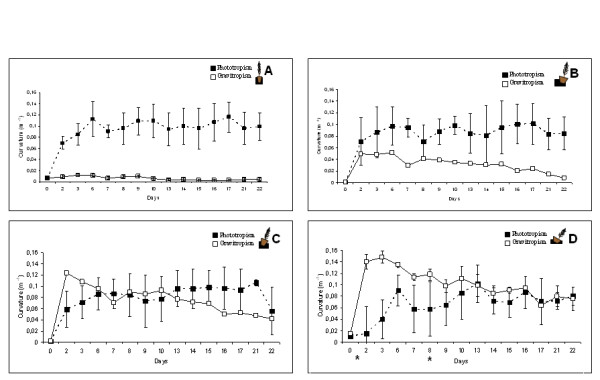
**Stem reorientation with regards to light and gravity**. Stem reorientation was measured with regard to the light source (phototropism: black squares, dotted line) and the vertical plane (gravitropism: white squares, solid line) in plants A) vertical (0°) or inclined at A) 0°, B) 15°, C) 30° and D) 45°. Asterisks indicate sampling dates for proteomic analysis. Data are means ± standard deviation.

In all seedlings, shoot apical movement was detected only after 2 h. In vertical plants (not inclined, 0°) and inclined at 15° or 30°, the stem apex oriented towards the light source (black squares in Figure 2A, B and 2C) at the same speed. In vertical plants (Figure 2A), no significant gravitropic movement occurred. Although the apex then reoriented with regard to the vertical plane in plants inclined at 15° (Figure 2B, white squares), the curvature towards the light source (black squares) was always significantly greater, even after 22 days. In plants inclined at 30° (Figure 2C) and 45° (Figure 2D), the apex first reoriented in the vertical plane (white squares), before then turning towards the lateral light source after 24 h (see additional file 1: movie #1). Gravitropism was more pronounced during the first 2 or 3 days of plant inclination, and then tended to decrease, regardless of leaning angle. From 6 days onwards, the stem curvature in the apical region of these plants was not significantly different with regard to light and verticality, i.e. apices reoriented towards both light and the vertical axis at similar speeds. Apical shoot curvature was highly variable in response to unilateral light exposure, whereas little variability was observed with regard to gravistimulation.

#### Re-orientation of the basal region

In the basal region of the seedlings, no significant changes in stem angle occurred, with regard to either light or gravity, even after 22 days (additional file 2: Supplemental Figure F1A, B).

In plants where the apex had been removed, no phototropic or gravitropic response in the upper part of the shoot was observed during the first 24 h (see additional file 3: movie #2).

### Proteomic analysis of photo- and gravi-tropic responses

#### Source of protein variation

**Figure 3:**
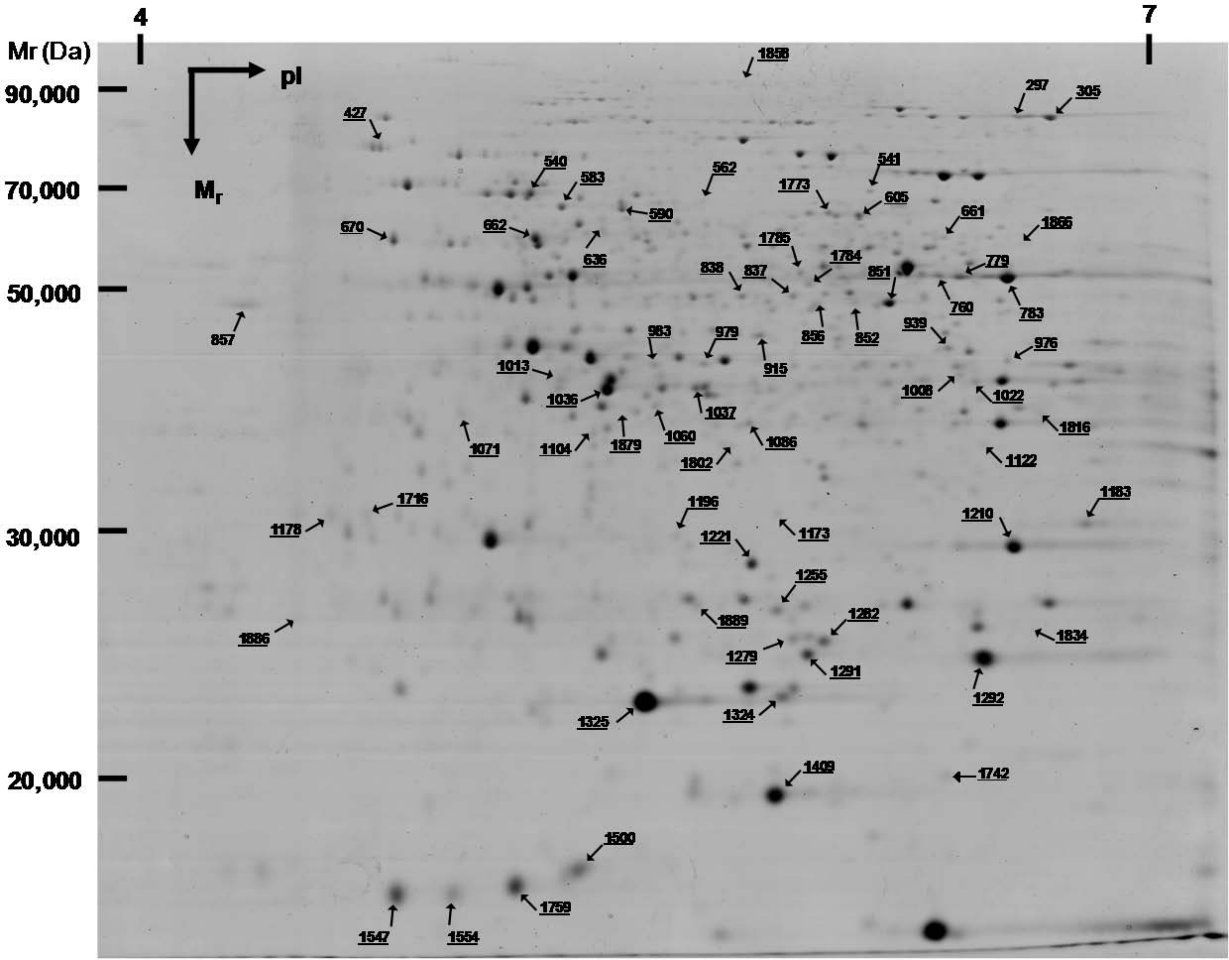
**2-DE maps of the maritime pine apical shoot**. Proteins were extracted from vertical (0°) and inclined plants at 0° and 45°. Proteins that were identified are shown with arrows and numbered as in Supplementary Table 1.

Differential intensity was observed in 68 spots (Figure 3 for at least one effect (P < 0.005). While three spots (22, 8 and 7) showed only Time (T), inclination (I) or TxI effects, respectively, 23 spots displayed all the three effects (additional file 4: Supplemental Figure F2). Significant differences in protein abundance were detected for more spots than expected by chance alone (2.4 spots at a P-value of 0.005), showing that the two main factors (time or inclination) thus play important roles in protein synthesis regulation.

#### Samples and proteins clustering

The hierarchical clustering of the 12 samples (2 levels of inclination * 2 time points * 3 replicates, additional file 5: Supplemental Figure F3) showed that replicates clustered together, which indicated a good reproducibility of the 2DE technique. Samples inclined at 45° for 22 hr formed a first branch leading away from a second branch, which comprised samples taken after 22 hr at 0° and samples corresponding to 8 days of treatment at 0° and 45° lean. In term of protein clustering, three distinct sub-trees were identified (G1, G2, G3). The third group (the largest group) mainly comprised proteins up-regulated after 22 hr in plants inclined at 45°.

**Figure 4:**
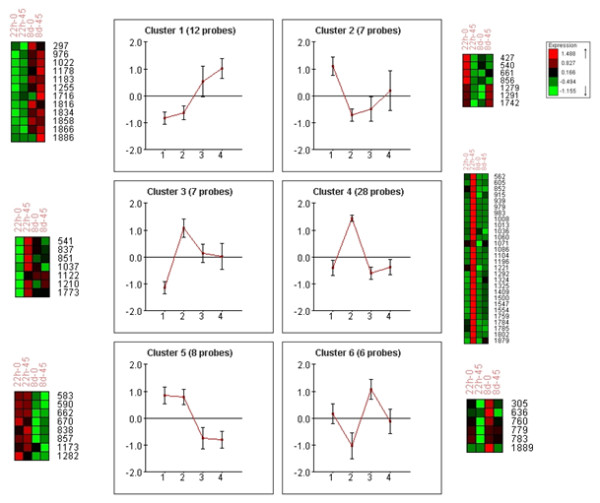
**Samples sub-clustering according to their accumulation profile**. Clustered mean protein accumulation profiles of differentially expressed proteins (1: 22 hr0°; 2:22 hr-45°; 3: 8d-0°; 4: 8d-45°). Clusters were obtained using the "K-means function" of Expander software on standardised data (mean 0 and standard variation 1). Error bars represent the standardised protein accumulation levels variation. Right and left panels display the list and protein accumulation profile of proteins belonging to each cluster (caption as in fig. 3).

Differentially abundant proteins were also clustered according to their expression profiles using the K-means algorithm. This analysis clustered the 68 spots into six groups (Figure 4), with a mean homogeneity of 0.913 and a mean separation score of -0.166. The protein accumulation profiles in each cluster were therefore highly homogeneous. The highest homogeneity was observed for cluster#4 (28 spots, i.e. 41% of the significant proteins). This cluster presented a remarkable signature, all proteins being consistently over-expressed in stems inclined at 45° after 22 hr. Most proteins of this cluster presented similar coefficients of determination for T, I and TxI effects. Protein profiles in cluster #3 (7 spots) resembled those of cluster #4, but the contrast between stems inclined at 45° for 22 hr and the other three treatments was less pronounced. Cluster #5 (8 spots) and to a less extent cluster #1 (12 spots) displayed very typical profiles with proteins over-expressed for 22 hr and 8 days, respectively, independent of the leaning angle, therefore presenting almost exclusively a T effect. Cluster #6 (6 spots) and cluster #2 (7 spots) presented a less clear pattern, although proteins of cluster #6 were systematically under-expressed in plants inclined for 22 hr and 8 days, and proteins of cluster #2 had a higher protein accumulation level after 22 hr in vertical at 0°.

The 68 differently expressed spots were manually cut from gels and characterized by LC ESI MS/MS. Detailed protein identification data, including peptide sequences, charge states and individual peptide scores were stored and available in the proticDB database [26]http://cbi.labri.fr/outils/Protic/public/PublicMAP.php. From this initial set of spots, i/we only considered those proteins presenting a single hit (therefore avoiding a mixture of proteins resulting from protein co-migration with similar electrophoretic properties or cross-contamination during the picking) identified with at least two peptides, and ii/removed spots with large inconsistencies between theoretical and observed isoelectric points and/or molecular weights.

**Figure 5:**
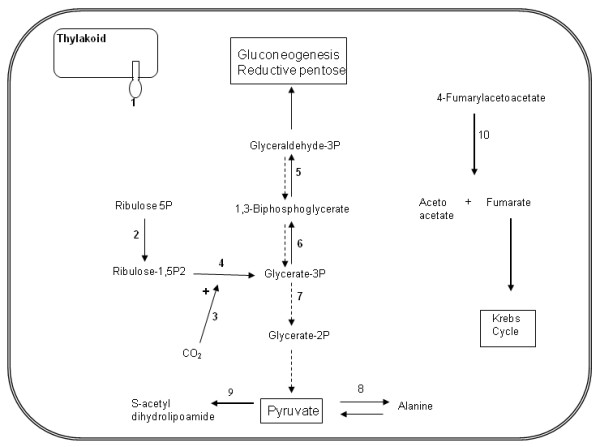
**Metabolic pathway based on differentially expressed proteins at 22 hr**. Enzymes recorded in this study are shown in numbers: 1, ATP synthase; 2, phosphoribulokinase hosphoribulokinase; 3, Rubisco activase; 4, Rubisco; 5, glyceraldehyde-3-phosphate dehydrogenase; 6, phosphoglycerate kinase; 7, phosphoglycerate mutase; 8, alanine aminotransferase; 9, pyruvate dehydrogenase; 10, fumarylacetoacetate. Full arrows (→) follows photosynthesis pathway. Dashed arrows (- - - - →) follows enzymes involved in glycolysis.

Finally, 48 spots (listed in additional file 6: Supplemental table S1) were kept for the biological interpretation of our results, including ten spots (#1183, #1210, # 1292, #1324, #1325, #1409, #1500, #1547, #1554, #1759) corresponding clearly to degradation products of Rubisco (a major soluble protein in all plants). The sequenced proteins spots were grouped according to their annotated functions. Several categories were identified, including primary metabolism, energy, cell rescue, defence, virulence, cell cycle, DNA processing, and response to biotic/abiotic stimuli. Most of the differentially expressed proteins belonged to "Energy" (39%), "Primary Metabolism" (29%) and "Cell, rescue and defence" (11%). Proteins of cluster #1 (Figure 5) included alcohol dehydrogenase (#976), glyceraldehyde phosphate dehydrogenase precursor (#1022), plastid lipid associated protein (#1178), and Rubisco (#1183). In cluster #5, some of the proteins identified were enolase (#838), phosphoglucomutase (#590), thiamine biosynthetic enzyme (#1173), and Rubisco (#670). Cluster #3 comprised alanine aminotransferase (#851), heat shock protein (#541), phosphoglyceromutase (#1773), and glutamine synthetase (#1037). In cluster #4, proteins corresponded to ATP synthase (#1196), adenosylhomocysteinase (#1785), pyruvate dehydrogenase E1 (#1071), phosphoglycerate kinase (#1104 and #1086), glyceraldehydes-3-phosphate dehydrogenase (# 1060), and degraded products of Rubisco large subunit (#1292, #1324, #1325, #1409, #1500, #1547, #1554, and #1759).

## Discussion

### Phenotypic response to light and gravity

Our experiment showed that depending on the initial angle of stem lean, plant response to unilateral irradiation differed. Apical phototropic reorientation occurred after 2 h, although such responses have been observed after only few seconds in Arabidopsis [27]. When initial stem leaning angle was zero (not inclined) or 15°, shoot tips turned preferentially towards the light and stem curvature towards the vertical axis was low in leaning trees. At 22 days, apices had almost finished their reorientation to the vertical. When initial stem lean was 30° or greater, shoot tips oriented with regard to the vertical axis before, turning towards the light source. After approximately 6 days, the degree to which stems maintained a given curvature was similar in both directions. Stem curvature over 22 d was not enough for stems inclined at 30° and 45° to return to 0°. On the contrary, stems were maintained at a given curvature after 7-9 days (Figure 2). Stem basal angle was also maintained at the original leaning angle. Therefore the older parts of stems of these seedlings did not exhibit a strong reoriention with regard to the vertical, which does not mean absence of biochemical response, especially subsequent compression wood formation occurs. Similar results were found by Ba et al [28] comparing reorientation strategies in young maritime and loblolly (*Pinus taeda*) pines. These authors found that different strategies for maintaining stems in a given spatial position exist between both species. Digby and Firn [29] discussed this phenomenon and determined that the angle, at which any part of an organ is maintained as a result of gravitropism, is controlled by developmental and environmental factors. This angle has been termed the 'gravitropic set-point angle' (GSA). Both the light environment and the initial gravitropic treatment can change the GSA. In our experiment, the light source was orthogonal, therefore plant orientation did not fully return to the vertical. Once the initial responses had occurred in plants, and equilibrium reached with regard to light and gravity, plant position in space was maintained. One of the only ways in which the GSA can be constantly changed, is by a repeated dynamic stimulation of the gravitropic response through, e.g. sporadic wind loading [30].

### Proteomic response to light and gravity

Inclining maritime pine seedlings triggers a stem response at the proteome level which can be reflected in plant morphology [4,31]. We were able to identify the differential protein accumulation in the stem apex. These differentially expressed proteins were clustered into three main groups (additional file 5: Supplemental Figure F3), or six different sub-clusters (Figure 4) depending on the clustering algorithm. Given the patterns of stem reorientation, the level of expression of each protein in the clusters and the type of effect (either Time (T) and Inclination (I) and/or T*I), we suggest that: (i) cluster #4 and to a lesser extent cluster #3 mainly contained proteins responding early on to gravitropism and therefore likely to be associated to the typical primary gravitropic response, (ii) clusters #1 and #5 comprised proteins responding independently of the bending angle and most probably are associated with a combined phototropic growth and developmental effect, (iii) cluster #6 was characterized mainly by proteins over-expressed in straight plants, therefore responding positively to phototropism and negatively to gravitropism, and (iv) cluster #2 comprised proteins with a strong interaction between T and I. This cluster also had a clear signature with regard to proteins under-expressed at 22 hr for inclined plants. Based on these observations, we hypothesized that clusters #3 and #4 contained early responding gravitropism associated proteins, whereas clusters #1 and #5 contained proteins whose expression was largely related to phototropic growth and development after 8 days of treatment. In the following section, we have focused the discussion on those proteins grouped in cluster #4 that were clearly up-regulated after 22 hr in inclined plants.

### Characterization of early responding gravitropic associated proteins

The proteome and the transcriptome of maritime pine have been studied for several years in adult trees and many genes and proteins have been reported to be involved in the secondary gravitropic response involving reaction wood formation [31-33]. To our knowledge, this study is the first to identify differentially expressed proteins in the primary response to stem bending at the apex of young seedlings. Based on protein function (additional file 6: Supplemental Table S1) we propose that the underlying molecular mechanisms involved in the gravitropic response necessitates energy supply and the synthesis of carbohydrate polymers. Therefore, the most important group of proteins identified were those related to "energy", and "metabolism" (Figure 5). Some of these proteins, e.g. ATP synthase, aminotransferase, aldolase and heat shock protein have been identified as differentially expressed with regard to the gravitropic stimulus in roots [34,35], but none have been related to the primary gravitropic response in the stem apex.

### Energy/photosynthesis related proteins

The present proteomic study identified not only proteins already reported as involved in gravitropism, e.g. glyceraldehyde-3 phosphate dehydrogenase (G3PDH, spots #939, #979, #983, #1060), Rubisco large subunit (#1292, #1324, #1325, #1409, #1500, #1547, #1554, #1759, #1210), but also, several new proteins associated with gravitropism such as ATP synthase (#1196), phosphoglycerate mutase (#1773), phosphoribulokinase (#1013), Rubisco activase (#1036) and phosphoglycerate kinase (PGK, #1104, #1086). ATP synthase was detected as differentially expressed which could be explained by the high requirement of energy needed to quickly reorient in space. In an inclined poplar hybrid (*Populus tremula × Populus alba*) this protein was also identified suggesting that its over expression can be related to an energy production or in response to oxidative stress [36]. The accumulation of G3PDH and PGK involved in glucose degradation and the production of energy also suggest an active metabolism in the production of pyruvate, ATP and other intermediates. However, the presence of Rubisco activase, a chaperone of Rubisco, also suggests regulation of Rubisco activity and the hydrolysis of ATP [37].

The large sub-unit of Rubisco, was responsible for the most differentially accumulated proteins, where a total of 13 spots, which included 10 degraded products of Rubisco were identified. Three of those spots (#760, #779, # 783) were found to be up-regulated after 22 hr on straight plants (see cluster #6 in figure 5), the observed and theoretical M*r *were similar, suggesting that these proteins were involved in the phototropic response. The remaining nine spots (listed above) were up-regulated after 22 hr on inclined plants (cluster #4). Observed M*r *ranged from 14 to 25 kDa, indicating that the degradation of Rubisco occurs in leaning plants after 22 hr. Rubisco has been found to be degraded in plants subjected to abiotic stresses [38-42]. These studies suggested that the degradation products of Rubisco were reutilized for the synthesis of proteins in response in to an imposed stress. In our study, given the rapidity of the phenotypic response (occurring only 2 hours after stem leaning - see additional file 1: Supplemental movie #1) it is likely that the demand for the synthesis of novel structural proteins could only be met by the recycling of amino acids from degraded Rubisco.

### Metabolism related proteins

The group of differentially expressed proteins which are involved in primary metabolism can also provide substrate for the synthesis of secondary metabolites. The 8 proteins identified from this group comprised: NADP-dependent D-sorbitol-6-phosphate dehydrogenase (#1122), carbonic anhydrase (spots #1221), adenosylhomocysteinase (spot #1785), adenosine kinase (spot #1879), pyruvate dehydrogenase (#1071), fumarylacetoacetase (#915), alanine aminotransferase (#851), and glutamine synthetase (#1037). The latter three enzymes are involved in the synthesis of amino acids and their over-expression may indicate the requirement of new proteins. A concerted modulation of alanine and glutamate metabolism exists in stressed plants [43]. Alanine aminotransferase catalyses the translocation of amino groups between alanine and pyruvate, maintaining the balance between carbon and nitrogen metabolism [44]. Pyruvate generated in the cytoplasm could be mobilized into the mitochondria where the enzyme pyruvate dehydrogenase catalyzes oxidative decarboxylation generating acetyl-CoA.

Adenosine kinase catalyzes the phosphorylation of AMP having adenine and ATP as substrates. Sorbitol-6-phosphate dehydrogenase is a key enzyme in sorbitol biosynthesis where it catalyzes the NADPH-dependent reduction of glucose-6-phosphate to sorbitol-6-phosphate. With regard to carbonic anhydrase, this enzyme may serve a protective role, which results in a complex with Rubisco in the thylakoids' outer membranes, preventing metal toxicity [45]. Finally, adenosylhomocysteinase is involved in the methionine metabolism generating homocysteine.

## Conclusions

The apical stems of maritime pine seedlings inclined at 45° rapidly reorient with regard to the vertical axis, whereas little or no response is observed at the stem bases of the same plants. This strong primary gravitropic response is accompanied by a modification of the proteome of the stem apex, consisting of an accumulation of energy and metabolism associated proteins. Intense degradation of Rubisco LS and the accumulation of amino acid biosynthesis related proteins may be required to meet this demand.

## Methods

### Plant material and experimental design

Maritime pine seeds (*Pinus pinaster *Aït.) collected from a forest stand in the Aquitaine region (France) were germinated on a mixture of sand/peat/bark (1:1:1). Seedlings were planted into 4L pots and kept in a greenhouse at 25°C. Five month-old plants were then moved to a growth chamber (8 h dark/16 h light at 25°C) and pots were inclined at three different angles from the vertical (0°, 15°, 30° and 45°). Plants were illuminated with halogen lamps (314 - 494 μmol.m^-2^.s^-1^) and lamps were situated in the horizontal direction, perpendicular to the direction of lean in inclined plants (Figure 1). Each tree was placed in a compartment which prevented light contamination from any lamps nearby. Compartments were built from black cardboard on wooden frames and air was able to circulate freely (Figure 1). A total of 13 plants were used per leaning angle.

### Analysis of the tropic response

#### Short term response

We followed the kinetics of shoot reorientation during the first 24 h after inclining plants by automatically taking photographs with a digital camera (Canon powershot A95) every 5 minutes. Images were then compiled into a movie using Paint Shop Pro v7 (Jasc software, USA). These images allowed us to determine: i/the speed of plant reorientation and whether there was a preferential direction towards light or the vertical axis and, ii/which portion of the plant was responding to these stimuli.

To test whether the initial tropic responses were still observed in plants where the apex had been removed, we decapitated the main stem and branches of two individuals by removing the top 5 mm of shoot tissue with scissors.

#### Long term response

**Figure 6:**
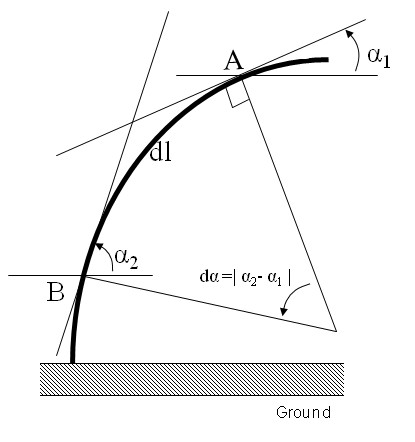
**Experimental design to determine global curvature**. The global curvature was determined by drawing a tangent between the base and the apex of the stem. Two points, A and B were then chosen on the stem and tangents drawn through these points with regard to the horizontal. The angle between the tangent and the horizontal was then measured. *dl *is the distance between A and B, following the curve of the stem and *dα *is the difference of angles between the tangent at A and the horizontal and the tangent at B and the horizontal.

**Figure 7:**
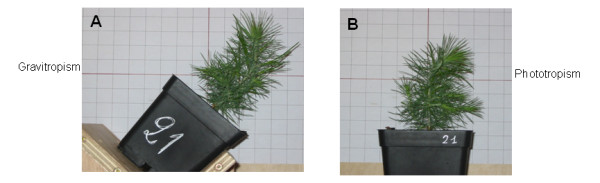
**Plant phenotype after light and gravitropic treatment**. A) Gravitropic stem curvature (0.075 m^-1^) in a plant leaning at 45° after 22 days of inclination. B) Phototropic stem curvature (0.080 m^-1^) in a vertical plant irradiated with unilateral light after 22 days of treatment.

Photographs were taken (in both the direction of lean and light) at 1 or 2 day intervals from day 0 to day 22, on four plants per treatment, in order to follow the kinetics of stem re-orientation over a period of 3 weeks. Stem curvature was used as a measure of reorientation and was calculated by drawing tangents onto the photographs of each plant using the image analysis software IMAQ TM version Builder (v1.0). The global curvature *C *was deduced from the angle variation *dα *between two tangents to the stem centreline. These tangents were taken at points A and B located near the apex and the base of the stem respectively (Figure 6). The global stem curvature is then given by:

(1) $$C=\frac{\pi d\alpha }{180dl}$$

where *dl *is the curvilinear distance between A and B. Using the same photographs, we also measured stem basal displacement from the vertical axis. These data provided us with a simple description of stem leaning angle at the plant base (Figure 7).

#### Choice of samples for molecular analysis

The observations of stem reorientation were used to define where and when plant material should be collected for molecular analysis. It was decided to sample plants for the proteomic analysis after 22 h and 8 days to evaluate both the early and late responses to gravitropic stimulation. To take into account the receptive and responding cells, only the apical (i.e. a whorl of young needles) and subapical (the stem without the euphylls and pseudophylls) regions of the shoot were sampled. To compare extreme responses to light and gravity within the apical region, we analyzed plants inclined at 0° and 45° only.

#### Protein extraction, quantification and separation

For the molecular analysis, the apices of five plants taken randomly from four conditions (0 and 45° after 22 hours and 8 days after treatment) were sampled, pooled, placed in liquid nitrogen and stored at -80°C before protein extraction. The samples were pooled in order to increase the amount of extracted proteins. Finally, three groups of five plants each were taken for each condition and were used as biological replicates. Samples were taken at the same time at both sampling dates, i.e. date, i.e. 3 h after dawn.

When studying the proteome at an individual level, a common drawback in the data analysis is that the distribution of the studied variables (protein abundance measured as spot volume) is unknown. This shortcoming limits the statistical tools that can be applied to non-parametric methods, which do not make any assumptions on the distribution of data and on their variance homogeneity. A solution to circumvent this problem is to use pooled samples. In this case, each spot volume will actually represent the average of the given variable (protein abundance) for the pooled random sample which, according to the central limit theorem, will be normally distributed between samples. This method allows the use of parametric statistics for the comparison of the new defined variables (protein abundance averages).

Total proteins were extracted from 500 mg of fresh tissue following the method originally described by Damerval *et al *[46] and modified by Gion *et al *[32]. Protein amount was quantified on six replicates, using a modified Bradford assay described by Ramagli and Rodriguez [47]. The mean concentration was then calculated and used to load 300 μg of proteins on IPG-strips. The protocol described by Gion *et al *[32] was used to separate the proteins according to their Ip and Mw. The gels were stained using Coomassie brilliant blue G250 (Biorad, Hercules, CA).

#### Image acquisition, spot detection and statistical analysis

Stained gels were digitized using a scanner and the LabScan software (Amersham Biosciences, Uppsala, Sweden). First, a calibration with a grey scale was necessary to transform grey levels into optical density (OD) values for each pixel of the gel picture. A colloidal blue calibration (Labscan) with a grey scale was used. Image analysis was performed using the Image Master 2D-Elite software (IM2D: Amersham Bioscience). The wizard detection method proposed by the software was used to detect the spots. Automatically detected spots were then manually checked, and some manually added or removed. Following the detection procedure, the volume for each spot corresponded to a gross value. In order to eliminate the background from this gross value, the mode of non spot of IM2 D was used.

Replicated gels were matched within a folder in order to attribute a common spot identity for the same spots derived from different images. For this, we used the automatically matching options of IM2 D. After visual checking of the matching, the IM2 D software was used to build a master gel. For each sample, when a protein was detected in all of the replicates, it was automatically added to the master gel, thus creating a reference map for this tissue. Normalized volumes were finally obtained using the total spot volume normalization procedure of IM2 D. The following linear model was then applied to each spot:

(2) $${\text{Y}}_{\text{ijkl}}=\mu +{\text{T}}_{\text{j}}+{\text{I}}_{\text{k}}+({\text{T}}_{\text{j}}\times {\text{I}}_{\text{k}})+\varepsilon_{\text{ijkl}},$$

where Y_ijkl _is the normalized volume of spot i (i = 1-486), at time j (j = 1-2, i.e. 22 hr *vs*. 8 days), at inclination k (k = 1-2, *i.e*. 0° *vs *45°). Three technical replicates (l = 1-3) were performed. ANOVAs were performed using R (R Development Core Team, 2004) with a type I sum of squares to obtain the main and interaction effect determination coefficients. For each of the 486 spots detected by two-dimensional gel electrophoresis, two-way analysis of variance allowed the detection of those proteins showing significant time (T), inclination (I) and/or interaction (TxI) effects.

#### Data clustering of differentially expressed proteins

Centred-reduced data of proteins showing at least one significant effect were analyzed using two types of clustering methods implemented in the Expander software [48]. A first analysis was performed using hierarchical clustering (Euclidian distance, Unweighted Pair*-*Group Method, UPGM algorithm) to group the spots and the samples. A second analysis was carried out using the K-means algorithm [49] to group proteins showing similar profiles along the four tested conditions. In K-means clustering, reference vectors (here set at 6) are initialised randomly, and proteins are partitioned to their most similar reference vector. Each reference vector is recalculated as the average of the protein that mapped to it and this step is repeated until convergence, i.e. all proteins map to the same partition on consecutive iteration.

#### In-gel protein digestion

Coomassie blue stained protein spots were manually excised from the gels and washed in H_2_O/MeOH/acetic acid (47.5/47.5/5) until destaining. The solvent mixture was removed and replaced by acetonitrile (ACN). After shrinking off the gel pieces, ACN was removed and gel pieces were dried in a vacuum centrifuge. Gel pieces were rehydrated in 8 ng/μL trypsin (Sigma-Aldrich, St. Louis, MO) in 50 mM NH_4_HCO_3 _and incubated overnight at 37°C. The supernatant was removed and the gel pieces were shaken for 15 min in 50 mM NH_4_HCO_3 _at room temperature. This second supernatant was pooled with the previous one, and a H_2_O/ACN/HCOOH (47.5/47.5/5) solution was added to the gel pieces for 15 min. This step was repeated twice. Supernatants were pooled and concentrated in a vacuum centrifuge to a final volume of 25 μL. Digests were finally acidified by addition of 1.2 μL of acetic acid (5% v/v) and stored at -20°C.

#### Nanospray LC- MS/MS and data analysis

Peptide mixtures were analyzed by on-line capillary chromatography (LC Packings, Amsterdam, The Netherlands) coupled to a nanospray LCQ ion trap mass spectrometer (ThermoFinnigan, San Jose, CA). Peptides were separated on a 75 μm inner diameter × 15-cm C18 PepMap column (LC Packings). The flow rate was set at 200 nL/min. Peptides were eluted using a 5-65% linear gradient of solvent B in 30 min (solvent A was 0.1% formic acid in 2% acetonitrile, and solvent B was 0.1% formic acid in 80% acetonitrile). The mass spectrometer was operated in positive ion mode at a 2 kV needle voltage and a 38 V capillary voltage. Data acquisition was performed in a data-dependent mode consisting of, alternatively in a single run, a full scan MS over the range m/z 300-2000 and three full scan MS/MS of the three most intense ions in the precedent MS spectra. MS/MS data were acquired using a 2 *m/z *units ion isolation window, a 35% relative collision energy, and a 5 min dynamic exclusion duration. Peptides were identified with SEQUEST through the Bioworks 3.2 interface (Thermo-Finnigan, Torrence, CA, USA) using the 45,934 Tentative Contigs (TCs) of The Gene Index Databases, TIGR (The Institute for Genomic Research, Rockville MD) http://compbio.dfci.harvard.edu/tgi. When mixtures of proteins were found, their relative quantities were estimated using the Pepquant function of SEQUEST Software. Identified proteins were classified following the functional categories defined by the Munich Information Center for Protein Sequences (MIPS, http://mips.gsf.de).

## Authors' contributions

RH carried out the protein studies, statistical analysis, selection of differentially expressed proteins, and drafted the manuscript. CK carried out protein extraction, and 2 D gel analysis, plant inclination experiments and apical curvature measurements. CL participated in protein extraction, 2 D gel analysis, spot isolation and protein sequencing. EHMB participated in the experimental design, plant inclination trials and curvature measurements. AS conceived the study, participated in the design and helped to draft the manuscript. FS carried out video recording and coordinated the inclination experiment. TF contributed to the experimental design and the apical curvature analysis. SC participated in the protein sequencing and analysis. CP conceived the study, participated in its design and coordination and helped to draft the manuscript. All authors read and approved the final manuscript.

## Supplementary Material

Additional file 1 **Supplemental movie #1**. Apex reorientation on inclined stem. Movie showing apex reorientation during the first 24 hours after plant inclination. Light was supplied laterally. Click here for file

Additional file 2 **Supplemental Figure F1. Basal reorientation with regards to light and gravity**. Basal stem leaning angle in all treatments with regard to the vertical (y) axis in response to A) perpendicular illumination and B) gravity, over 22 days. Click here for file

Additional file 3 **Supplemental movie #2. Stem reorientation on decapitated plants**. Movie showing stem reorientation during the first 24 hours after inclination on decapitated plants. Light was supplied laterally. Click here for file

Additional file 4 **Supplemental Figure F2. Venn diagram on significant spots**. Venn diagram of the 68 significant spots (P < 0.005). Click here for file

Additional file 5 **Supplemental Figure F3. Samples clustering according to their protein distance**. Clustering of samples and technical replicates within samples, according to their protein distance (Euclidian distance of centered - reduced data, UPGM algorithm). The scale bar adjacent to each dendogram represents the distance measurement used Expander software algorithm [(1-Pearson correlation)/2]. The colour scale bars represent the relative standardized content of proteins. For each spot, data were standardized to give a mean of 0 and standard deviation of 1. Click here for file

Additional file 6 **Supplemental table S1. List of identified spots**. Spots identified from the databank and considered for biological interpretation. Click here for file
