# Supplementary figures and images for: (Not) Keeping the stem straight: a proteomic analysis of maritime pine seedlings undergoing phototropism and gravitropism
Source: BMC Plant Biol. 2010 Oct 6;10:217. doi: 10.1186/1471-2229-10-217 (PMC3017815; doi:10.1186/1471-2229-10-217)

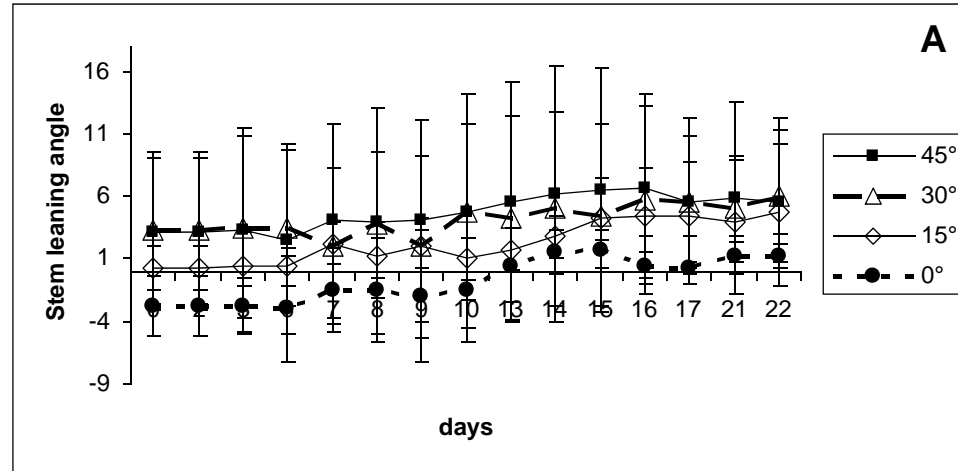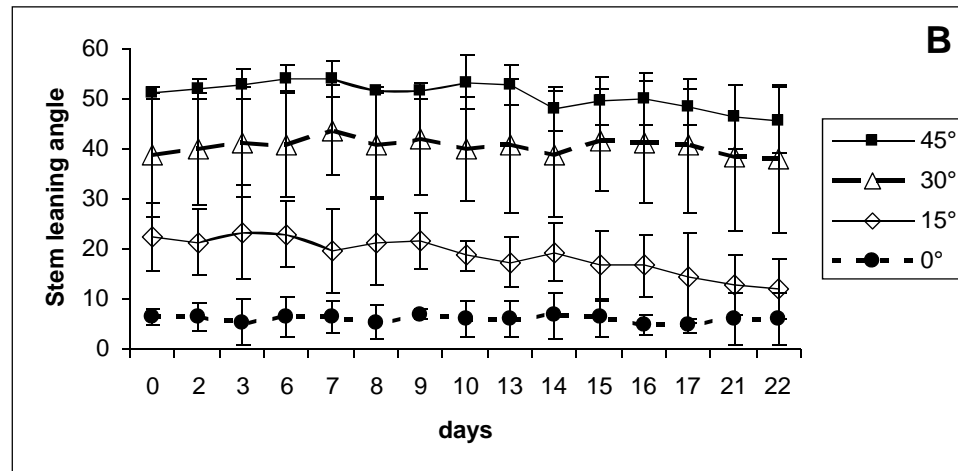

Supplement: Additional file 2 — Supplemental Figure F1. Basal reorientation with regards to light and gravity. Basal stem leaning angle in all treatments with regard to the vertical (y) axis in response to A) perpendicular illumination and B) gravity, over 22 days. [file 1471-2229-10-217-S2.PDF]

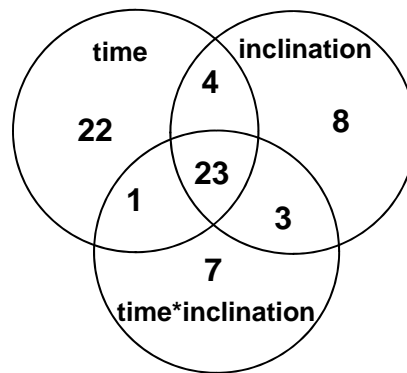

Supplement: Additional file 4 — Supplemental Figure F2. Venn diagram on significant spots. Venn diagram of the 68 significant spots (P < 0.005). [file 1471-2229-10-217-S4.PDF]

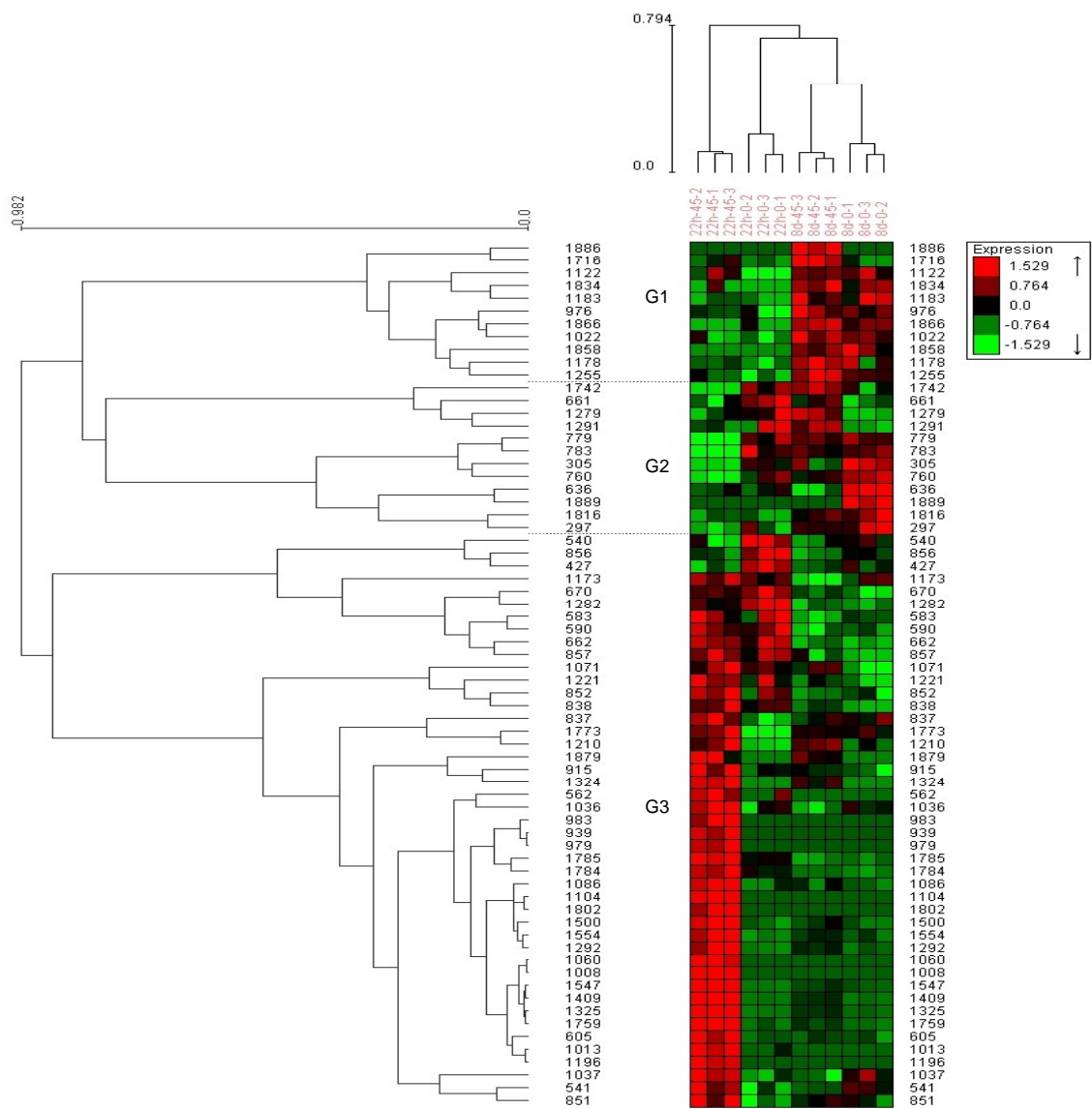

Supplement: Additional file 5 — Supplemental Figure F3. Samples clustering according to their protein distance. Clustering of samples and technical replicates within samples, according to their protein distance (Euclidian distance of centered - reduced data, UPGM algorithm). The scale bar adjacent to each dendogram represents the distance measurement used Expander software algorithm [(1-Pearson correlation)/2]. The colour scale bars represent the relative standardized content of proteins. For each spot, data were standardized to give a mean of 0 and standard deviation of 1. [file 1471-2229-10-217-S5.PDF]
